# Do children with ADHD symptoms become socially isolated? Longitudinal within-person associations in a nationally-representative cohort

**DOI:** 10.1192/j.eurpsy.2023.280

**Published:** 2023-07-19

**Authors:** K. N. Thompson, J. C. Agnew-Blais, A. G. Allegrini, B. T. Bryan, A. Danese, C. L. Odgers, T. Matthews, L. Arseneault

**Affiliations:** 1 Social, Genetic and Developmental Psychiatry Centre, King’s College London; 2 Department of Psychology, Queen Mary University; 3 Department of Clinical, Educational and Health Psychology, University College London, London, United Kingdom; 4 Social Science Research Institute, Duke University, Durham, United States

## Abstract

**Introduction:**

Social isolation in childhood can be detrimental to physical and mental health. Children with neurodevelopmental disorders, such as attention deficit hyperactivity disorder (ADHD), may be particularly at risk for becoming socially isolated. Similarly, isolated children have limited opportunities to observe, model, and learn age-appropriate interpersonal interactions with other children which could increase ADHD behaviours.

**Objectives:**

This study examined longitudinal associations between ADHD symptoms and social isolation across childhood. We tested the direction of this association across time, while accounting for pre-existing characteristics, and assessed whether this association varied by ADHD presentation, informant, sex, and socioeconomic status.

**Methods:**

Participants included 2,232 children from the Environmental Risk (E-Risk) Longitudinal Twin Study. ADHD symptoms and social isolation were measured at ages 5, 7, 10, and 12. We used random-intercept cross-lagged panel models to assess the directionality of the association across childhood.

**Results:**

Children with increased ADHD symptoms were consistently at increased risk of becoming socially isolated later in childhood, over and above stable characteristics (β=0.05-0.08). These longitudinal associations were not bidirectional; isolated children were not at risk of worsening ADHD symptoms later on. Children with a hyperactive ADHD presentation were more likely to become isolated, compared to an inattentive presentation. This was evident in the school setting, as observed by teachers, but not by mothers at home.

**Conclusions:**

Our findings highlight the importance of enhancing peer social support and inclusion for children with ADHD, particularly in school settings. We add explanatory value over and above traditional longitudinal methods as our results represent how individual children change over time, relative to their own pre-existing characteristics.

**Disclosure of Interest:**

None Declared

